# Evaluation of hypertension knowledge and its association with medication adherence among hypertensive patients attending primary health centers: a cross-sectional study from eastern Saudi Arabia

**DOI:** 10.3389/fpubh.2024.1378561

**Published:** 2025-01-13

**Authors:** Ahmad Homoud Al-Hazmi, Abdullah Dhoimi Mureed Alanazi, Ashokkumar Thirunavukkarasu, Nasser Saleh Alriwely, Mmdoh Mohammad F. Alrais, Alreem Barghash S. Alruwaili, Mona Saleh Alnosairi, Amnah Ibrahim Alsirhani

**Affiliations:** ^1^Department of Family and Community Medicine, College of Medicine Jouf University, Sakaka, Saudi Arabia; ^2^Department of Compliance, Ministry of Health, Arar, Saudi Arabia; ^3^College of Medicine Jouf University, Sakaka, Saudi Arabia; ^4^Family Medicine Resident, Ministry of Health, Tabuk, Saudi Arabia

**Keywords:** hypertension, knowledge, adherence, health center, complications, Saudi Arabia

## Abstract

**Background and aim:**

The global healthcare system acknowledged the crucial role of disease knowledge in health outcomes and improving quality of life among patients with chronic disease. A lack of adequate knowledge and understanding of hypertension, its symptoms, and available treatments can lead to poor treatment outcomes. The present study aimed to determine the level of hypertension knowledge and associated factors among hypertensive patients. Furthermore, we evaluated the correlation between levels of knowledge and medication adherence among them.

**Methods:**

The present study was carried out among 406 hypertensive patients attending different primary health centers in Hafr Al Batin, Saudi Arabia. Participants’ hypertension-related knowledge was evaluated using the validated hypertension knowledge–level scale, and adherence practice was evaluated using the medication adherence and refill scale. We categorized the knowledge score into low, medium, and high, according to Bloom’s criteria. We applied Spearman’s correlation test to find the strength and direction of the correlation between hypertension-related knowledge and medication adherence. Furthermore, we used binomial logistic regression analysis to find the associated factors of the low hypertension-related knowledge among the patients.

**Results:**

Of the studied patients, only 10.3% demonstrated a high level of knowledge, and the highest knowledge levels were observed in the domains of complications ( $\bar{x\;}$ = 4.39, standard deviation [SD] = 1.20) and lifestyle ( $\bar{x\;}$ = 3.13, SD = 0.69), while knowledge about drug compliance ( $\bar{x\;}$ = 0.62, SD = 0.98) was the lowest. A statistically significant positive correlation was observed between knowledge and adherence regarding hypertension (rho = 0.268, *p* = 0.001) among study participants. We observed that marital status (*p* = 0.032), income (*p* = 0.042), and absence of chronic diseases (*p* = 0.001) are associated factors for low hypertension-related knowledge.

**Conclusion:**

The study findings highlight a moderate level of knowledge about hypertension among patients, with significant gaps in drug compliance understanding. The positive correlation between knowledge and medication adherence underscores the need for better hypertension education at primary health centers. Furthermore, it is recommended that future prospective studies be conducted within various cultural contexts.

## 1 Introduction

Hypertension is a chronic medical condition characterized by elevated blood pressure. The World Health Organization (WHO) has estimated that about 1.3 billion people suffer from hypertension worldwide, and nearly half of them are not aware of their hypertension status (1). Hence, it is increasingly emerging as a worldwide public health concern, encompassing a significant portion of public health financial allocations (2, 3). The prevalence of hypertension exhibits variation across different countries, with a notable upward trend observed in developing nations (4, 5). People with uncontrolled hypertension are at high risk of experiencing heart failure, renal failure, atherosclerosis, and stroke (6, 7). Hypertension, however, is preventable, treatable, and can be managed through medication (2, 8).

A higher risk of negative consequences, such as hospitalization and higher healthcare expenses due to problems, is seen in individuals with poor adherence to antihypertensive drugs compared to those with strong adherence. So, medication adherence is necessary to achieve blood pressure control. Medication adherence refers to the percentage of patients who take their prescribed medications as their treating physician recommends (9, 10). Peacock and Krousel-Wood report that only 50% of people with “chronic diseases medication,” for instance, hypertensive patients, adhere to their medication regimen as prescribed (11). Another research conducted in 2022 among hypertensive adult patients (18+ years) attending primary health centers (PHC) in the Kingdom of Saudi Arabia (KSA) shows that only 36% of the hypertensive patients had high adherence to the treatment (12). In contrast, a comprehensive survey conducted in the United States, including an extensive sample of 24 million individuals diagnosed with hypertension, has revealed that a notable proportion, specifically about one-third, exhibited nonadherence to their prescribed drug regimen (13).

The global healthcare system acknowledges the crucial role of disease knowledge in health outcomes and improving quality of life among patients with chronic disease. Health professionals should provide patients with information about the benefits of taking their drugs and how to ensure that they are taking them as prescribed, which is essential to help patients control their blood pressure in the long term and reduce the risk of developing complications of hypertension (14, 15). An integrated approach to managing hypertension and increasing literacy levels among hypertensive patients and healthcare professionals is essential. The physicians and other healthcare practitioners must then collaborate to ensure success in helping patients avoid complications related to high blood pressure (16, 17). One way to achieve this is through a shared decision-making (SDM) model. Shared decision-making refers to a collaborative decision-making process in which all involved in the decision-making process have a stake in the outcome. The SDM model is particularly effective in managing chronic conditions like hypertension, where long-term adherence to treatment plans is crucial. By involving patients in the decision-making process, they are more likely to understand the importance of their medication regimen, lifestyle modifications, and the potential consequences of nonadherence (18, 19). Educating hypertensive patients is necessary to make them aware of the condition and the available medications so that they can make informed decisions about their care (17, 20). This collaborative approach can improve patient satisfaction, adherence to treatment, and overall health outcomes (21, 22).

A lack of adequate knowledge, awareness, and understanding of hypertension, its symptoms, and available treatments can lead to poor treatment adherence. Furthermore, a good knowledge about disease improves self-management, which is critical for patients to self-manage their situations (23). Studies have consistently shown that a lack of knowledge is a significant factor associated with poor adherence, and patients with a lack of knowledge are more likely to discontinue medications for several reasons (24, 25). Moreover, some studies have shown that knowledge regarding hypertension is generally low among hypertensive patients in KSA. Among those with some knowledge, there was a significant correlation between knowledge level, blood pressure control, and medication adherence (26, 27).

In the KSA, healthcare is delivered at four levels: PHCs, general hospitals, specialty hospitals, and medical cities. PHCs are integral to the healthcare system and provide essential services, including the diagnosis, management, and follow-up of chronic diseases such as hypertension. Continuous monitoring of epidemiological characteristics related to hypertension, such as knowledge of hypertension in terms of risk factors, complications, treatment compliance, and lifestyle factors among the patients attending PHCs, is critical to planning and implementing an intervention program to address the knowledge gap, eventually to improve the medication adherence and health of the individuals with hypertension. Furthermore, the sociocultural and demographic characteristics of each region of KSA, including Eastern Province, differ highly. Hence, there is an absolute necessity for region-specific data and evaluation. Understanding the local context and challenges in this specific region can inform tailored interventions and improve hypertension management practices in this region. Considering these factors, we aimed to assess the level of hypertension knowledge and associated factors among the patients attending different PHCs of Hafr Al Batin. Furthermore, we assessed the correlation between levels of knowledge and medication adherence among them.

## 2 Participants and methods

### 2.1 Study design

The present study was an analytical cross-sectional study that was performed from June 2023 to December 2023.

### 2.2 Study setting

The present survey was conducted among the patients attending different PHCs of Hafr-Al Batin. Hafr Al-Batin is in the Eastern province of KSA, with a total population of approximately about 400,000. These centers are critical in diagnosing, treating, and educating patients about hypertension, thereby playing a crucial role in mitigating its complications and improving the overall health outcomes of the patients. There are 73 PHCs in this region, and the present study selected 10 PHCs randomly to recruit hypertensive patients for the current survey. In order to recruit hypertensive patients over a period of time, we restricted five patients per day in each PHC.

### 2.3 Sample size estimation and sampling method

We used the World Health Organization (WHO) sample size formula (n = z^2^pq /e^2^) to estimate the required number of adults to participate in the present survey. The WHO formula uses the principles of Cochran’s sample size calculation method, a commonly used method to calculate the minimum required sample size in a cross-sectional study (28). During estimation, we considered 50% of expected adequate knowledge (p), q = 1-p, 95% of the confidence interval (*z* = 1.96), and 5% of the margin of error (d). After carefully calculating the above-stated values, we concluded that 384 participants are required for the survey (rounded to 390). These required participants were selected from 10 randomly selected PHCs. In this study, we applied a non-probability consecutive sampling method, a type of convenience sampling where participants were consecutively selected based on their availability and meeting the inclusion criteria until the required sample size was achieved. Furthermore, we restricted our data collection per day to a maximum of 10 participants to ensure that the data is collected over a period of time.

### 2.4 Inclusion and exclusion criteria

We included all adult-confirmed hypertensive patients who were on antihypertensive medications for a minimum of 3 months and attended the outpatient clinics of the PHCs from the Hafr Al-Batin region. The 3 months duration was determined by the research team through a focus group discussion considering the guidelines of follow-up visit (29, 30). We excluded pediatric patients, pregnant women, patients from other regions, and patients who were unwilling to participate.

### 2.5 Data collection procedure

The present research followed the ethical principles of the Declaration of Helsinki during the entire study duration. We initiated the data collection after obtaining ethical clearance from the research committee, the Ministry of Health, Hafr Al-batin (approval no: 134), and other necessary approvals from the concerned authorities. After briefing the survey to the hypertensive patients and obtaining informed consent, we gave the participants the Arabic version of the data collection tool (Google form). This was on the day of their visit to the health center, and the data was filled in the data collectors’ electronic devices. The data collection tool is a standard, pretested, and validated tool adapted from previous literature (12, 31–33). The first section inquired about participants’ background and health-related aspects: age, gender, income, occupation, education, presence of other comorbidities, duration since the first diagnosis of hypertension, and the number of medications taken for hypertension. The second part asked about participants’ hypertension-related knowledge using the hypertension knowledge–level scale (HK-LS) (32, 34). The HK-LS consists of 22 items to investigate participants’ knowledge regarding six subscales, including definition (two items), medical treatment (four items), drug compliance (four items), lifestyle (five items), diet (two items), and complications (five items). The correct answer was given 1 point, and the wrong answer, not sure, was given zero. Participants’ overall knowledge was based on summing up the correct responses to these items. Therefore, the overall knowledge scores ranged between 0 and 22. We further categorized them into low (less than 60% of total score), medium (60 to 80% of total score), and high (more than 80% of total score). The research team determined these categories according to Bloom’s criteria, which is used in several studies (35–37). The final section inquired about medication adherence and refill practices among the selected participants and consisted of 10 questions (12, 31). Hypertensive patients responded with a range of answers, from never (score 4) to always (score 1). The scores of each item were summed up to get the total scores for further analysis. The higher scores indicate a higher adherence practice. Initially, we pretested the tool among 30 adult hypertensive patients. All pilot study patients were informed that the data collection tool was simple and easy to understand. The Cronbach alpha values for the knowledge and adherence sections that were obtained through pilot study were 0.79 and 0.83, respectively.

### 2.6 Statistical analysis

The research team downloaded the Excel sheet from Google Form and exported it to the Statistical Package for Social Sciences (SPSS) version 23.0 for data entry, coding, and analysis. We presented the descriptive data as frequency, proportion, mean/median, and standard deviation/interquartile range. The normality assumption of the data was analyzed through the Shapiro–Wilk test. The test revealed that both hypertension-related knowledge scores (statistical value = 0.971, *p* < 0.001) and medication adherence scores (statistical value = 0.982, *p* < 0.001) did not meet the normality assumption. Hence, we applied Spearnman’s correlation test (non-parametric test) to find the correlation between knowledge and medication adherence practice. Furthermore, we used the binary logistic regression analysis (enter method) to find the factors for the knowledge categories. In this regression model, we calculated the odds ratio with a 95% confidence interval after adjusting with other covariables of the study. A *p*-value less than 0.05 will be considered a statistically significant association.

## 3 Results

The majority of participants were male (60.3%), residing in urban areas (80.3%), and currently married (89.2%). Nearly half of the participants belonged to the age group of 45 to 60 years, comprising 48.8% of the total respondents. Most participants had a university degree or higher (75.1%). Almost half of the participants were employed in the government sector (47.8%). The vast majority of participants had an income exceeding 7,000 SAR (82.8%). Most participants were non-smokers (84.2%). Over half of the participants had hypertension for 5 years or above (54.4%). One-third of the participants reported having chronic diseases (33.3%; Table 1).

**Table 1:** Socio-demographic characteristics of the study participants (*n* = 406).

Parameters	Frequency	Proportion
Gender		
Male	245	60.3
Female	161	39.7
Residence status		
Urban	326	80.3
Rural	80	19.7
Marital status		
Currently married	362	89.2
Single	44	10.6
Age groups, year		
Less than 45	105	25.9
45 to 60	198	48.8
More than 60	103	25.4
Education status		
Up to high school	101	24.9
University and above	305	5.1
Employment status		
Government sector	194	47.8
Private sector / Business	153	37.7
Unemployed	59	14.5
Income		
Less than 5,000 SAR	40	9.9
5,000 to 7,000 SAR	30	7.4
More than 7,000 SAR	336	82.8
Smoking status		
Nonsmoker	342	84.2
Smoker	64	15.8
Duration of hypertension		
Less than 1 year	30	7.4
1–5 years	155	38.2
5 years and above	221	54.4
Chronic diseases		
Yes	135	33.3
No	271	66.7

The study participants demonstrated a relatively high awareness of fundamental aspects of hypertension, such as the importance of daily medication intake (94.3%) and the necessity of lifelong medication adherence (83.0%). However, a considerable proportion of study participants believe that high blood pressure only occurs with aging (17.5%) and that lifestyle changes can negate the need for medication (18.2%). Moreover, there were notable gaps in knowledge regarding dietary habits, as a significant number thought it was permissible for hypertensive individuals to consume salty foods (12.8%) or drink alcohol (15.8%). Furthermore, the highest levels of wrong answers were observed on the knowledge of the statements: people with high blood pressure should only take their medications when they feel tired and unwell (90.9%), people with high blood pressure can eat salty foods as long as they take their medications regularly (87.2%), and type of food intake such as frying food (86.7%). The majority of participants have a medium level of knowledge (51 to 75%), and only 10.3% demonstrate a high level of knowledge (above 75%; Tables 2, 3).

**Table 2:** Participants’ responses to hypertension knowledge-level scale.

Items	Correct answer		Wrong answer	
	n	%	n	%
Systolic or diastolic hypertension is an indication of high blood pressure	349	86.0	57	14.0
Diastolic hypertension (number II) alone is also considered as an indicator of high blood pressure	212	52.2	194	47.8
Medications prescribed to treat high blood pressure should be taken daily	383	94.3	23	5.7
People with high blood pressure should only take their medications when they feel tired and unwell.	37	9.1	369	90.9
People with high blood pressure should take their medications throughout their lives.	337	83.0	69	17.0
People with high blood pressure should take their medications in a way that makes them feel comfortable	54	13.5	351	86.5
If medications can control high blood pressure, there is no need to change the patient’s lifestyle and daily behaviors.	55	13.3	352	86.7
High blood pressure occurs with aging, so there is no need to take treatment in such a case	71	17.5	335	82.5
If a person with high blood pressure succeeds in changing his lifestyle and healthy behaviors in his daily life, then there is no need to continue taking treatment	74	18.2	332	81.8
People with high blood pressure can eat salty foods as long as they take their medications regularly.	52	12.8	354	87.2
People with high blood pressure can drink alcohol	64	15.8	342	84.2
People with high blood pressure should not smoke	382	94.6	22	5.4
People with high blood pressure should eat vegetables and fruits constantly	386	95.1	20	4.9
Frying foods is the best way to prepare meals for hypertensive patients	54	13.3	352	86.7
Boiling or grilling foods is the best way to prepare meals for hypertensive patients	384	94.6	22	5.4
The best types of meat that are suitable for hypertensive patients are white meat	362	89.2	44	10.8
The best types of meat that are suitable for hypertensive patients are red meat	101	24.9	305	75.1
If not treated, high blood pressure can cause early death.	354	87.2	52	12.8
If not treated, high blood pressure can cause cardiovascular diseases such as thrombosis.	374	92.1	32	7.9
If not treated, high blood pressure can cause strokes.	367	90.4	39	9.6
If not treated, high blood pressure can cause kidney failure.	329	81.0	77	19.0
If not treated, high blood pressure can cause eye and vision problems.	356	87.7	50	12.3

**Table 3:** Level of Knowledge related to hypertension among participants (*n* = 406).

Variable	Frequency	Proportion
Low (<60%)	94	23.2
Medium (60 to 80%)	270	66.5
High (> 80%)	42	10.3

The overall mean knowledge score for hypertension among the 406 participants was 12.66 (SD = 2.41), indicating a moderate level of knowledge. Participants demonstrated the highest knowledge levels in the domains of complications ( $\bar{x\;}$ = 4.39, SD = 1.20) and lifestyle ( $\bar{x\;}$ = 3.13, SD = 0.69), while knowledge about drug compliance ( $\bar{x\;}$ = 0.62, SD = 0.98) was the lowest (Table 4).

**Table 4:** Participants’ responses to knowledge-domain wise.

Variable	All participants; *n* = 406	
	Mean	Standard deviation
Total knowledge (Domain all)	12.66	2.41
Knowledge about definition of hypertension (D1)	1.38	0.69
Knowledge about medical treatment (D2)	2.00	0.61
Knowledge about drug compliance (D3)	0.62	0.98
Knowledge about lifestyle (D4)	3.13	0.69
Knowledge about diet (D5)	1.14	0.41
Knowledge about complications (D6)	4.39	1.20

Regarding antihypertensive medication adherence, 51.5% of the patients never forget to take their medicines, and more than two-thirds (71.4%) of the patients never stopped their medicines even when their blood pressure is controlled. Similarly, about 55% of patients always refill their antihypertensive medications well in advance before they finish (Table 5).

**Table 5:** Participants’ responses to medication adherence scale (*n* = 406).

Variable	Never	Once in a while	Majority of the times	Always
	n (%)	n (%)	n (%)	n (%)
How often do you forget to take your medication to control blood pressure?	209 (51.5)	175 (43.1)	14 (3.4)	8 (2.0)
How frequently do you choose not to take your medication?	256 (63.1)	127 (31.3)	18 (4.4)	5 (1.2)
Do you change the medicine dose or number of tablets without physician consultation?	231(57.1)	150 (36.9)	19 (4.7)	5 (1.2)
Do you stop taking medications on the day you feel comfortable?	230 (56.7)	153 (37.7)	16 (3.9)	7 (1.7)
Have you ever stopped taking your medications without telling your doctor because of your perceived side effects?	275 (67.7)	100 (24.6)	22 (5.4)	9 (2.2)
How frequently do you forget to take medicine due to forgetfulness?	227 (55.9)	129 (31.8)	35 (8.6)	15 (3.7)
How often do you stop medicines when your blood pressure is controlled?	290 (71.4)	83 (20.4)	15 (3.7)	18 (4.4)
How frequently have you stopped taking blood pressure medicine while you are sick due to other acute illnesses such as flu?	288 (70.9)	95 (23.4)	14 (3.4)	9 (2.2)
Have you ever been annoyed by adhering to the treatment plan, as some patients feel discomfort taking it daily?	294 (72.4)	90 (22.2)	16 (3.9)	6 (1.5)
How frequently do you refill well in advance before the blood pressure medicine finishes?*	8 (2.0)	48 (11.8)	127 (31.3)	223 (54.9)

*Reverse scoring.

A statistically significant positive correlation was observed between knowledge and adherence regarding hypertension (rho = 0.268, *p* = 0.001) among study participants (Table 6).

**Table 6:** Correlation of knowledge about hypertension with adherence.

Variable	rho/ *p*-value
Knowledge–Adherence	0.268 (0.001)

In the univariate analysis, several socio-demographic factors were identified as predictors of knowledge about hypertension. Participants aged 45 to 60 years (crude odds ratio [COR] = 0.339, 9% confidence interval [CI] = 0.180–0.639, *p* = 0.001), females (COR = 2.058, 95% CI = 1.290–3.282, *p* = 0.002), those residing in rural areas (COR = 4.687, 95% CI = 2.771–7.927, *p* = 0.001), employed in the private sector/business (COR = 2.206, 95% CI = 1.192–4.084, *p* = 0.012), unmarried individuals (COR = 6.245, 95% CI = 3.240–12.038, *p* = 0.001), those with high income, non-smokers (COR = 0.299, 95% CI = 0.124–0.716, *p* = 0.001), and those with 1 to 5 years of hypertension duration (COR = 0.224, 95% CI = 0.102–0.492, *p* = 0.001) were significantly associated with the hypertension related knowledge. After adjusting for confounding factors in the multivariate analysis, marital status (adjusted odds ratio [AOR] = 2.538, 95% CI = 1.086–5.932, *p* = 0.032), income (AOR = 0.364, 95% CI = 0.138–0.963, *p* = 0.042), and absence of chronic diseases (AOR = 0.491, 95% CI = 0.260–0.932, *p* = 0.001) remained significant predictors of knowledge about hypertension (Table 7).

**Table 7:** Univariate and multivariate regression analysis for the predictors of knowledge regarding hypertension.

Variables	Total	Univariate analysis					Multivariable analysis		
		Low; *n* = 94	Medium / High; *n* = 312	Unadjusted odds Ratio (OR)	95% CI	*p*-value	Adjusted; OR*	95% CI	*p*-value
Age (in years)									
Less than 45	105	42	63	Ref			Ref		
45 to 60	198	33	165	0.339	(0.180–0.639)	0.001	0.590	(0.239–1.458)	0.253
More than 60	103	19	84	1.131	(0.607–2.108)	0.698	0.992	(0.473–2.080)	0.983
Gender									
Male	245	44	201	Ref			Ref		
Female	161	50	111	2.058	(1.290–3.282)	0.002	1.435	(0.816–2.522)	0.210
Residence status									
Urban	326	55	271	Ref			Ref		
Rural	80	39	41	4.687	(2.771–7.927)	0.001	1.837	(0.916–3.684)	0.087
Education status									
Up to high school	101	30	71	Ref			Ref		
University and above	305	64	241	0.628	(0.378–1.045)	0.073	0.528	(0.275–1.012)	0.054
Employment status									
Government sector	194	46	148	Ref			Ref		
Private sector / Business	153	24	129	2.206	(1.192–4.084)	0.012	0.638	(0.263–1.545)	0.319
Unemployed	59	24	35	3.686	(1.871–7.260)	0.001	1.199	(0.493–2.917)	0.689
Marital status									
Currently married	362	68	294	Ref			Ref		
Single	44	26	18	6.245	(3.240–12.038)	0.001	2.538	(1.086–5.932)	0.032
Income									
Less than 5,000 SAR	40	24	16	Ref			Ref		
5,000 to 7,000 SAR	30	12	18	0.139	(0.070–0.278)	0.001	0.364	(0.138–0.963)	0.042
More than 7,000 SAR	336	58	278	0.313	(0.143–0.685)	0.004	0.519	(0.202–1.331)	0.172
Smoking status									
Nonsmoker	342	88	254	Ref			Ref		
Smokers	64	6	58	0.299	(0.124–0.716)	0.007	0.525	(0.200–1.382)	0.192
Duration of hypertension									
Less than 1 year	30	16	14	Ref			Ref		
1–5 years	155	33	122	0.224	(0.102–0.492)	0.001	0.960	(0.337–2.735)	0.939
5 years and above	221	45	176	0.945	(0.570–1.566)	0.827	1.595	(0.844–3.014)	0.151
Chronic diseases									
Yes	135	72	199	Ref			Ref		
No	271	22	113	0.538	(0.317–0.915)	0.002	0.492	(0.260–0.932)	0.001

*Enter method was applied in regression analysis. Adjusted with other covariables of the study.

## 4 Discussion

Hypertension poses a significant public health burden worldwide. In KSA, its prevalence is alarming, with one in four adults estimated to have the condition (38). To effectively combat this challenge, understanding the knowledge about hypertension and adherence to hypertensive medication of hypertensive individuals is crucial. The present study delves into the knowledge and medication adherence landscape among patients attending various primary health centers in Saudi Arabia, offering valuable insights for tailored interventions and improved health outcomes. The findings from this study underscore the ongoing challenge of managing hypertension in Saudi Arabia, particularly in the Eastern region.

Overall, participants’ knowledge about hypertension was moderate (mean 2.66), which is similar to research conducted in Lebanon (39), Sri Lanka (24), Indonesia (25), and Pakistan (40). However, specific knowledge domains revealed notable disparities. Participants demonstrated a strong understanding of complications (mean 4.39) and lifestyle modifications (mean 3.13), aligning with the research findings of Buang NFB et al. (41) and Alshammari SA et al. (42). Conversely, a significant knowledge gap was identified in our study regarding drug compliance (mean 0.62), similar to the concerns raised in studies from India (43) and Pakistan (44). The current study highlights the persistent knowledge gaps that could contribute to suboptimal health outcomes.

The present study also reports that the participants have poor knowledge in the domains “definition and diet.” This finding is similar to the study done by Al Zahrani S et al. (26) and Jankowska-Polańska B et al. (45). Low scores in understanding definitions, diet, and drug compliance may be attributed to various factors. These could include unclear communication from healthcare providers, insufficient awareness of the significance of medication adherence, or cultural beliefs influencing medication use. Exploring these contributing factors can guide the development of targeted interventions. Tailored interventions like communication training for health care providers and behavioral change communication to the community will address these root causes and empower individuals and communities for improved health outcomes. The present study found several domains related to medication adherence and proper diet. These results emphasize the importance of the SDM model, as many patients were unaware of the importance of medication adherence even if they felt well and the importance of proper dietary methods. Furthermore, as a process of SDM, healthcare professionals must provide patients with comprehensive information about the benefits of medications even if the patients are feeling well, the role of lifestyle changes in managing hypertension, and the importance of regular monitoring and follow-up. Additionally, addressing patients’ concerns, preferences, and values is crucial in developing a personalized treatment plan that patients are more likely to follow (18, 46, 47).

In our study, about half of the participants responded as “always” in most of the medication adherence scale items. This aligns with recent findings by Amer M et al. (40), Lee EK et al. (48) Shi S et al. (49), and Gavrilova A et al. (50), high prevalence of nonadherence to antihypertensive medication globally with non-western countries showing even higher rates (48). These stark figures underscore the persistent challenge of medication nonadherence in chronic disease management, particularly in hypertension, affecting over 1.3 billion people globally, as highlighted by the World Health Organization in 2023 (51). This warrants further investigation into the specific factors influencing adherence patterns within low-adherence populations. Exploring modifiable factors, such as financial barriers, medication complexity, and patient-provider communication, could inform targeted interventions to improve adherence and ultimately optimize clinical outcomes, especially in the context of hypertension, where uncontrolled blood pressure can lead to devastating consequences like heart attacks and strokes. Our study provides specific insights into these gaps, such as the limited understanding of drug compliance and diet, which are crucial for effective hypertension management. These insights emphasize the need for targeted educational interventions at primary health centers to enhance patients’ knowledge and adherence to treatment regimens.

Similar to the research findings by Amer M (40) and Paczkowska A et al. (52), the present study shows that knowledge about hypertension was found to have a statistically significant positive correlation with medication adherence with a coefficient of 0.268, indicating a positive association between medication adherence and knowledge about hypertension. Patients with an adequate knowledge of hypertension may better perceive the need for regular drug adherence in managing their disease, resulting in a better dedication to following prescribed treatment regimens. Furthermore, the correlation coefficient of 0.268 suggests a positive relationship between hypertension awareness and medication adherence, highlighting the importance of patient education in improving positive health behaviors. This can lead to better blood pressure control and overall health outcomes, reducing the burden on the healthcare system due to complications arising from poorly managed hypertension.

We found that the knowledge level of the participants about hypertension is influenced by factors such as education, income, marital status, and the presence of chronic conditions. Individuals who are married, have chronic illnesses, have lower incomes, and are less educated are more likely to have a lack of knowledge about hypertension. These results are consistent with studies conducted in Poland (52), Malaysia (41), Saudi Arabia (38), and Lebanon (39). These warrants targeted intervention, especially for people of lower socio-economic status, to effectively manage this significant global health problem. Hence, we can decrease the associated complications and healthcare expenditure (53, 54). In agreement with recommendations from previous global studies, the present study offers a road map for creating proper interventions to increase awareness of hypertension and medication adherence. Culturally tailored health promotion activities that address specific knowledge gaps and target high-risk groups and include social aspects of health are vital. These programs must actively address the fears and myths surrounding the illness in addition to merely providing information. Furthermore, integrating community-based interventions and encouraging self-management abilities can enable people to take charge of their health and enhance long-term results worldwide. Additionally, integrating regular counseling sessions during follow-ups in PHCs could help reinforce the importance of medication adherence and lifestyle modifications.

We have carried out this survey with an adequate sample size and a validated Arabic questionnaire. Nonetheless, the readers should consider some of the essential constraints of this research findings. Firstly, we assessed the knowledge gap among hypertensive patients in a unique sociocultural setting. Hence, the conclusions assessed in this study may not be applicable to other regions. Secondly, the limitations of cross-sectional studies, such as the lack of ability to identify the causal association, need to be kept in mind. Next, the study faced limitations due to the non-probability consecutive sampling method, which may have introduced selection bias. Additionally, the higher proportion of male participants reflects sociocultural restrictions, affecting generalizability. Finally, some other biases, such as recall and self-report, cannot be ruled out.

## 5 Conclusion

We found that the majority of the participants had either low or medium knowledge of hypertension. Furthermore, a positive correlation was observed between hypertension-related knowledge and medication adherence. The results emphasize the need for better hypertension education at the primary health centers. It also emphasizes the need for culturally sensitive interventions that fill in knowledge gaps and provide people with the tools they need to take charge of their health. These issues can be effectively addressed by adopting targeted interventions for the high-risk population and the prevalence of hypertension in Saudi Arabia, enabling people to properly manage their health and enhance their quality of life. To contribute to a comprehensive understanding of this global challenge, it is recommended that future prospective studies be conducted to examine the factors influencing drug adherence, lifestyle modifications, and healthcare expenditure within a variety of cultural contexts.

## Data Availability

The raw data supporting the conclusions of this article will be made available by the authors, without undue reservation.
